# Effects of thyroid dysfunction on hematological parameters: Case controlled study

**DOI:** 10.1016/j.amsu.2020.07.008

**Published:** 2020-07-11

**Authors:** Sawer Sabri Ahmed, Ayad Ahmad Mohammed

**Affiliations:** a Department of Medial Laboratory Technology, Shekhan Technical College of Health, Duhok Polytechnic University, Duhok, Kurdistan Region, Iraq; b Department of Surgery, College of Medicine, University of Duhok, DUHOK, Kurdistan Region, Iraq

**Keywords:** Thyroid dysfunction, Erythrocytes, Anemia, Blood parameters

## Abstract

**Introduction:**

The thyroid gland has a very important role in hematopoiesis, blood disorders are frequently seen in patients with thyroid disorders. Thyroid hormones have direct effect on blood parameters by stimulating erythrocytes precursors and indirectly by enhancing erythropoietin production.

**Patients and methods:**

This is a case-control study which included 300 subjects who were grouped to 3 equal groups as a control, hypothyroidism, and hyperthyroidism groups. Patients with inherited or acquired red cell abnormalities, those receiving treatment for thyroid disorder or anemia, patient with chronic diseases, aged <12 years, pregnant ladies and patients unwilling to participate in the study were excluded.

**Results:**

The mean age of patients is 40.72 years, and females constituted 60.7% of cases. The analyses showed a significant difference the RBC, HB, MCV, MCHC, RDW, and WBC (P values 0.000, 0.000, 0.001, 0.012.0.002, and 0.027) respectively, while platelets showed no significant correlation (P value 0.08). The univariate analyses showed that RBC, the HB, and the WBC were the most severely affected parameters (Sig. 0.000, 0.000, and 0.005) respectively.

**Conclusion:**

The study concluded that females are more affected by thyroid disorders than males and the peak age is at the forties, thyroid dysfunction affect all blood parameters except platelets.The follow up of patients with thyroid disorders should include the complete blood count and patients diagnosed with anemia should be evaluated for thyroid disorders before iron therapy. Cases of anemia that resist treatment should be investigated for the possibility of thyroid dysfunction.

## Highlights

• The thyroid gland has a very important role in hematopoiesis.
• Females are more affected by thyroid disorders than males.
• Thyroid hormones directly stimulate erythrocytes precursors and indirectly by enhancing erythropoietin production.

## 1 Introduction

The thyroid gland has a very important role in the body metabolism in general including the hematopoiesis. Blood disorders are frequently seen in patients with thyroid disorders because thyroid hormones have a very crucial role in the proliferation and the metabolism of red blood cells and all other blood elements [1,2].

Abnormalities of thyroid function have a variety of clinical spectrums and it is a common clinical problem, many patients presented with subclinical derangements of the thyroid functions and are diagnosed on the basis of thyroid hormone evaluation or abnormal results of other investigations including blood parameters or lipid abnormalities, cardiac dysfunction, atherosclerosis, and many other clinical manifestations [[3], [4], [5], [6], [7]].

Anemia is a common clinical problem, its incidence in the general population may reach up to 10% in some parts of the world and it is most common females in the child-bearing age and the elderly population. Anemia is defined as a reduction in the number of red blood cells (RBC) or hemoglobin (Hb), this will result in reduction in the ability of the blood to carry oxygen to body tissues. According to the World Health Organization (WHO) recommendations, anemia is diagnosed when the Hb level is < 12.0 g/dL for women and <13.0 g/L for men. Normocytic anemia is defined as a mean corpuscular volume (MCV) between 80 and 100 fl, microcytic anemia is diagnosed as MCV below 80 fl, and macrocytic anemia by an MCV above 100 fl [[8], [9], [10]].

Thyroid hormones have a direct effect on the blood parameters by stimulating the precursors of the erythrocytes and indirectly by enhancing erythropoietin production [10].

Patients with thyroid abnormalities may have low iron levels which affect the hemoglobin levels, also they may have reduced levels of both folate and B12 which have been detected in up to 25% of patients, this eventually affects the blood parameters including the hemoglobin and the RBCs, other causes of anemia may include bone marrow suppression and other associated comorbid diseases [4].

Different types of anemia might be seen in patients with thyroid abnormalities, iron deficiency anemia is the commonest type of anemia in those patients, while microcytic or macrocytic anemia occur to a lesser extent [10].

Many authors have reported the correlation between the anemia and thyroid dysfunction, they estimated that more than 50% of patients have blood abnormalities. Many cases have subclinical hypothyroidism and they may present with abnormalities of blood parameters particularly anemia [11].

Anemia in patients with thyroid dysfunction is not only attributed to nutritional deficiency, but also due to reduction of thyroid hormones, this will result in lack of the stimulation of the erythrocyte precursors in the bone marrow, decrease in the oxygen supply to different tissues, and decrease in the erythropoietin level [11,12].

The aim of this study is to evaluate the effect of various types of thyroid function abnormalities on different blood parameters and comparing them to a group of apparently healthy individuals to evaluate any possible correlation between the levels of different blood parameters and different types of thyroid function abnormalities.

**Patients and methods:** This is a case-control study which were conducted in Azadi Teaching Hospital/Laboratory department in Duhok city, Kurdistan region, Northern Iraq.

The sample was collected between the periods from November 2018 to January 2019. About 300 subjects were enrolled in the current study and were grouped to 3 equal groups, including 100 patients with hypothyroidism, 100 patients with hyperthyroidism and 100 patients with normal thyroid and complete blood counts as control group. Two ml of Ethylenediaminetetraacetic acid (EDTA) anticoagulated blood and three ml of whole blood were taken from these subjects under fully aseptic condition for complete blood count (CBC) and thyroid function tests respectively. EDTA blood samples were put on mixer instrument for gentle mixing for 5 min. CBC was performed by Coulter counter. The hematological parameters which were studied include the white blood cells (WBC), red blood cells (RBC), hematocrit (HCT), hemoglobin (Hb), mean corpuscular volume (MCV), mean corpuscular hemoglobin (MCH), mean corpuscular hemoglobin concentration (MCHC), red cell distribution width (RDW), and Platelet counts. The other 3 mls of whole blood were put in gel tube, serum was prepared after centrifugation at 1000 gm and for 10 min, thyroid function tests including TSH, T3, T4 were performed based on electrochemiluminescence immunoassay (eCLIA) by Cobas 6000® (Roche Diagnostics).

Only patients with thyroid dysfunction TSH >5 iu/ml or TSH <0.25 were included as cases and those with normal thyroid function were included as control group.

Patients with known intrinsic red cell abnormalities (inherited or acquired), patients receiving treatment for thyroid disorder or anemia, patient with chronic diseases, patients aged < 12 years, pregnant ladies and patients unwilling to participate in the study were excluded. Patients with other diseases that may affect the blood parameters and those with evidence of nutritional deficiencies were also excluded. Data were collected before patients received treatment or any kind of intervention.

## 2 Statistical analysis

The data analysis were done and the expressions were displayed in terms of frequency, mean, median, and standard deviations. The correlations were done using the two-tailed t-tests and the univariate analysis. Statistical calculations were performed using the Statistical Package for Social Sciences (SPSS 25:00 IBM: USA).

## 3 Research registration

The research is registered according the World Medical Association's Declaration of Helsinki 2013 at the research registry at the 13th of December 2019, Research registry UIN: research registry 5645. Hyperlink to the registration page:

https://www.researchregistry.com/browse-the-registry#home/registrationdetails/5ece4d908f5e050015ccad38/

The work of this article has been reported in line with the STROCSS criteria [13].

## 4 Results

The mean age of patients who were involved in this study is 40.72 years, and females constituted 60.7% of cases. Most patients of them had normal blood parameters. Table 1.

**Table 1:** Shwoing the general chrecteristics of the patients enrolled in this study.

Main category (n = 300)	Subcategories	Frequency	Percentage
Age Range: 18-85		40.72	14.533
Gender	Male	117	39.0
	Female	182	60.7
Red blood cells	High	22	7.3
	Normal	269	89.7
	Low	9	3.0
Hb level	Normal	202	67.3
	High	4	1.3
	Low	94	31.3
Mean corpuscular volume	Normal	280	93.3
	Low	20	6.7
Mean corpuscular hemoglobin	Normal	269	89.7
	High	3	1.0
	Low	28	9.3
Mean corpuscular hemoglobin concentration	Normal	285	95.0
	Low	15	5.0
Red cell distribution width	Normal	284	94.7
	High	16	5.3
WBC count	Normal	287	95.7
	High	13	4.3
Platelets count	Normal	287	95.7
	High	5	1.7
	Low	8	2.7

Patients were categorized into 3 equal groups on the bases of thyroid function status into euthyroidisim, hypothyroidism, and hyperthyroidism groups. The analyses showed a significant difference in all the parameters between the three groups except the platelets which showed no significant correlation. Table 2.

**Table 2:** Cross tabulation table showing the comparison between different parameters which were studied among the three groups.

	Thyroid function status			Sig.
	Euthyroidisim	Hypothyroidism	Hyperthyroidism	
**Red blood cells**				**0.000**^a^
High	2 (2.0%)	3 (3.0%)	17 (17.0%)	
Normal	98 (98.0%)	90 (90.0%)	81 (81.0%)	
Low	0 (0.0%)	7 (7.0%)	2 (2.0%)	
**Hb level**				**0.000**^a^
Normal	94 (94.0%)	49 (49.0%)	59 (59.0%)	
High	0 (0.0%)	1 (1.0%)	3 (3.0%)	
Low	6 (6.0%)	50 (50.0%)	38 (38.0%)	
**Mean corpuscular volume**				**0.001**^b^
Normal	99 (99.0%	89 (89.0%	92 (92.0%	
Low	1 (1.0%	11 (11.0%	8 (8.0%	
**Mean corpuscular Hb concentration**				**0.012**^b^
Normal	99 (99.0%)	90 (90.0%)	96 (96.0%)	
Low	1 (1.0%)	10 (10.0%)	4 (4.0%)	
**Red cell distribution width**				**0.002**^b^
Normal	100 (100.0%)	89 (89.0%)	95 (95.0%)	
High	0 (0.0%)	11 (11.0%)	5 (5.0%)	
**WBC count**				**0.027**^b^
Normal	100(100.0%)	95(95.0%)	92(92.0%)	
High	0 (0.0%)	5 (5.0%)	8 (8.0%)	
**Platelets count**				0.080^a^
Normal	99 (99.0%)	92 (92.0%)	96 (96.0%)	
High	1 (1.0%)	2 (2.0%)	2 (2.0%)	
Low	0 (0.0%)	6 (6.0%)	2 (2.0%)	

a Fisher's Exact test.

b Pearson Chi-Square test.

The univariate analyses showed that RBC, the HB, and the WBC were the most severely affected parameters by the thyroid function status respectively, while the effect of the thyroid function status on the other parameters was less severe than the above mentioned ones. Table 3.

**Table 3:** Univariate regression table showing the most significant blood parameters which are related to thyroid function abnormalities.

Tests of Between-Subjects Effects					
Source	Type III Sum of Squares	df	Mean Square	F	Sig.
Age	.485	1	.485	.872	.351
Gender	.507	1	.507	.912	.340
RBCS	15.688	2	7.844	14.098	**.000**
Hb Value	15.915	2	7.957	14.301	**.000**
Mean corpuscular volume	.066	1	.066	.119	.730
Mean corpuscular Hb concentration	.178	1	.178	.321	.572
Red cell distribution width	1.919E-5	1	1.919E-5	.000	.995
WBC count	4.534	1	4.534	8.148	**.005**
Platelets count	1.367	2	.684	1.229	.294

a. R Squared = 0.887 (Adjusted R Squared = 0.881).

b. Dependent Variable: Thyroid function status.

## 5 Discussion

Anemia and other blood abnormalities are common in thyroid function abnormalities mainly hypothyroidism, and most patients are improved after thyroid hormone replacement and normalization of the thyroid function [4,14].

Thyroid abnormalities are common in the population being commoner in females in most studies, in our study there were female predominance (60.7%) similarly to most articles such as Dorgalaleh et al., but is lower than the findings of some other authors such as Preeti S. et al. and M. A. Iddah et al. who showed higher percentage in females and the male to female ratio was around 1:3.8 [[15], [16], [17]].

In our study we divided the enrolled individuals in to 3 equal groups, each group included 100 individuals, the first group were cases of hypothyroidism, and the other group were those having hyperthyroidism, these 2 groups were compared with an apparently healthy individuals as control group, generally it is estimated that cases of hypothyroidism are higher than hyperthyroidism when cases are taken randomly [16].

Many studies concluded that low hemoglobin levels are seen in patients with thyroid function derangement particularly hypothyroidism, in our study we concluded the same results and the level of hemoglobin was significantly associated with thyroid dysfunction (P value 0.000) and anemia was present in 31.3% of the patients in this study. Most authors recommend treatment with iron supplementation in those patients, patients who were treated with iron supplement alone without treatment with thyroxin showed lower response rates than combination therapy. Some population based studies confirmed that there is no significant association between anemia and thyroid dysfunction, this suggest that the results showed variation and this association is not constant [4,14,[17], [18], [19], [20]].

A univariate analysis was done and showed that the most affected blood parameters by thyroid dysfunction were the RBC, the Hb, and the WBC respectively, this may indicate that these parameters may be affected initially before the development of derangement of other parameters. Many studies showed that platelets are also affected by thyroid dysfunction, but in this study we found that platelets were the only parameters which showed no significant correlation with thyroid dysfunction (P value 0.080) [7].

A study which was done by Abdollah Jafarzadeh et al. showed that the MCV was significantly lower in patients with abnormal thyroid function compared to those with euthyroid status, this finding support the finding in our study, we found that the MCV was lower in patients with either hyper and hypothyroidism when compared to the normal individuals (P value 0.001) [2].

Red cell distribution width (RDW) represents the degree of RBC anisocytosis, it is increased in patients with iron deficiency anemia, B12 and folate deficiency, and thus it is affected by thyroid function derangement. In our study we detected a significant correlation between thyroid dysfunction and RDW (P value 0.002), however RDW may be affected by other clinical conditions such as inflammatory processes, cardiac diseases and rheumatoid arthritis [10,21,22].

Microcytic anemia is the commonest type of anemia seen in patients with thyroid dysfunction, MCV have been shown to be lower in patients with thyroid dysfunction compared to euthyroid individuals. In our study we found a very significant correlation between thyroid dysfunction and Hb and MCV (P values 0.000 and 0.001) respectively [10].

In a study which was done by Takahashi et al., who investigated different causes of anemia including macrocytic anemia, they found that hypothyroidism was one of the most important cause of anemia and abnormal red blood cell size [23].

Platelets are less affected by thyroid function status, this finding have been found in many other studies this may be due to the fact that platelets are non-nucleated and the have short life span with continuous rapid turnover [3].

The main limitations of this study is that larger number of patients are required to increase the accuracy of the findings and some population based data are required to determine the normal geographical and age related variation regarding the levels of the blood and thyroid test parameters.

## 6 Conclusion

The study concluded that females are more affected by thyroid disorders than males and the peak age is at the forties, thyroid dysfunction affect all blood parameters but platelets are less affected than other parameters indicating that thyroid hormones are very important for blood formation. The follow up of patients with thyroid disorders should include the complete blood count and all patients diagnosed with anemia should be evaluated for thyroid disorders before the start of iron therapy. Some cases of anemia that are resisting medical treatment or show very slow response to treatment may be due to thyroid function derangement.

## Funding

The author was only financial supporter of the study.

## Ethical approval

NA.

## Sources of funding

No source of funding other than the authors.

## Author contribution

Dr Sawer Sabri Ahmed did the data collection.

Study design, analysis, and writing is done by Dr Ayad Ahmad Mohammed.

Final approval of the manuscript is done by Dr Sawer Sabri Ahmed and Dr Ayad Ahmad Mohammed.

## Research registration unique identifying number (UIN)

Researchregistry5645

## If you are submitting an RCT, please state the trial registry number – ISRCTN

N/A.

## Guarantor

Dr Ayad Ahmad Mohammed.

## Provenance and peer review

Not commissioned, externally peer reviewed.

## Declaration of competing interest

No conflicts of interest present.
